# Sterol 27-Hydroxylase Polymorphism Significantly Associates With Shorter Telomere, Higher Cardiovascular and Type-2 Diabetes Risk in Obese Subjects

**DOI:** 10.3389/fendo.2018.00309

**Published:** 2018-06-13

**Authors:** Sofia Pavanello, Laura Angelici, Mirjam Hoxha, Laura Cantone, Manuela Campisi, Amedea Silvia Tirelli, Luisella Vigna, Angela Cecilia Pesatori, Valentina Bollati

**Affiliations:** ^1^Medicina del Lavoro, Dipartimento di Scienze Cardiologiche Toraciche e Vascolari, Università di Padova, Padova, Italy; ^2^Azienda Ospedaliera di Padova, Unità di Medicina del Lavoro, Padova, Italy; ^3^EPIGET – Epidemiology, Epigenetics and Toxicology Laboratory, Dipartimento di Scienze Cliniche e di Comunità, Università degli Studi di Milano, Milan, Italy; ^4^Dipartimento di Medicina Preventiva, Fondazione IRCCS Ca’ Granda Ospedale Maggiore Policlinico, Milan, Italy

**Keywords:** obesity, cholesterol, HDL, genotyping, cardiovascular diseases, diabetes mellitus type 2, insulin sensitive obese, telomere shortening

## Abstract

**Background/objectives:**

The pathologic relationship linking obesity and lipid dismetabolism with earlier onset of aging-related disorders, including cardiovascular disease (CVD) and type-2 diabetes (T2D), is not fully elucidate. Chronic inflammatory state, in obese individuals, may accelerate cellular aging. However, leukocyte telomere length (LTL), the cellular biological aging indicator, is elusively linked with obesity. Recent studies indicate that sterol 27-hydroxylase (CYP27A1) is an emerging antiatherogenic enzyme, that, by converting extrahepatic cholesterol to 27-hydroxycholesterol, facilitates cholesterol removal *via* high-density lipoprotein-cholesterol (HDL-C). We tested the hypothesis that obese subjects who carry at least three copies of CYP27A1 low-hydroxylation (LH) activity genome-wide-validated alleles (rs4674345A, rs1554622A, and rs4674338G) present premature aging, as reflected in shorter LTL and higher levels of CVD/T2D risk factors, including reduced HDL-C.

**Subjects/methods:**

Obese subjects from SPHERE project {*n* = 1,457; overweight [body mass index (BMI) 25–30 kg/m^2^] 65.8% and severe-obese (BMI > 30 kg/m^2^) 34.2%} were characterized for the presence from 0 to 6 LH-CYP27A1 allele copy number. Univariate and multivariable sex–age–smoking-adjusted linear-regression models were performed to compare CVD/T2D risk factors and biological aging (LTL) in relation to the combined BMI-LH groups: overweight-LH: 0–2, overweight-LH: 3–6, severe-obese-LH: 0–2, and severe-obese-LH: 3–6.

**Results:**

Higher LTL attrition was found in severe-obese than overweight individuals (*p* < 0.001). Multivariable model reveals that among severe-obese patients those with LH: 3–6 present higher LTL attrition than LH: 0–2 (*p* < 0.05). Univariate and multivariable models remarkably show that insulin resistance is higher both in overweight-LH: 3–6 vs overweight-LH: 0–2 (*p* < 0.001) and in severe-obese-LH: 3–6 vs severe-obese-LH: 0–2 (*p* < 0.0001), and HDL-C is lower in overweight-LH: 3–6 than overweight-LH: 0–2 (*p* < 0.05 and *p* < 001). Finally, most of the well-known (i.e., blood pressure, heart rate, waist to hip, triglycerides, and HDL-C) and novel CVD risk factors [i.e., inflammation markers (C-reactive protein, leukocytes, and chemoattractant protein-1), fibrinogen, and glucose homeostasis (i.e., insulin resistance, and glycated hemoglobin)] are substantially (*p* < 0.0001) altered in severe-obese-LH: 0–2 vs overweight-LH: 0–2, pointing to the fact that obesity leads to worsen the CVD/T2D risk factor profile.

**Conclusion:**

Our study supports evidence that CYP27A1 genetic characterization identifies persons at higher risk to develop CVD and T2D, on which better converge preventive measures, and opens new perspectives on mechanisms that link obesity with aging-related disorders.

## Introduction

The dramatic escalation of obesity and overweight prevalence, leading to earlier onset of aging-related cardiovascular disease (CVD), represents an emerging worldwide public health problem (1). This preventable disorder, often associated with cholesterol dismetabolism (2, 3) and aging-related diseases (4–6), is challenging to prevent and to treat, since the heterogeneous and complex causes, including interactions between genetic predisposition and environmental factors, are almost unknown (7).

The sterol 27-hydroxylase (CYP27A1) is a largely distributed mitochondrial P450 cytochrome enzyme, that, by converting extrahepatic cholesterol to 27-hydroxycholesterol (27OHC), the most abundant circulating oxysterol, primarily promotes its elimination. Extrahepatic CYP27A1, mainly expressed in the vascular endothelium (8) and in macrophages (9), in fact contributes to speed 27OHC elimination by the so-called “reverse transport,” from the periphery to the liver (10) where it is further converted to bile acids, the major cholesterol catabolic pathway. The flux of 27OHC from periphery to the liver is regarded as an emerging antiatherogenic mechanism flanking the classical HDL-dependent cholesterol reversed transport (11, 12). Lack in CYP27A1 sterol hydroxylation activity due to mutations in the *CYP27A1*, found in patients with cerebrotendinous xanthomatosis disease, determines cholesterol accumulation in the vascular endothelium and severe premature development of atherosclerosis even though normal serum cholesterol concentrations (13). In addition, mice, lacking in the CYP27A1 with the consequent lessening in 27OHC production, present a failing in estrogen-related cardiovascular protection (14). 27OHC, in fact, directly antagonizes the transcriptional and non-transcriptional functions of estrogen receptors in vascular endothelial and smooth muscle cells, flanking the cardioprotective effects of estrogens (15). Taken together, these data suggest a protective role of normal *CYP27A1* hydroxylation activity in the development of atherosclerosis and the consequent CVD risk. Reduced CYP27A1 activity has been also found in genome wide study to be mainly related to three *CYP27A1* SNPs (rs4674345 A>G, rs1554622 A>C, and rs4674338 G>A), each SNP presented similar contribution that explained 60–65% of its activity reduction (16). Likewise, these *CYP27A1* genetic polymorphisms could also diminish the cholesterol reverse transport, favor its accumulation in extrahepatic tissues, and therefore increase the individual atherogenic and CVD susceptibility.

Obesity may accelerate aging through chronic inflammation (17) and oxidative stress (18). Several observational studies have investigated the association between obesity and aging through assessment of telomere length in blood leukocytes (19, 20). The telomere in fact refers to terminal DNA–protein complexes at the chromosome, which primarily functions to maintain chromosomal stability (21). Telomere shortening in leukocytes (LTL) is regarded as marker of biological aging, but the association between obesity and LTL remains elusive (19, 20). Conversely, shorter LTL has been associated with higher risk of mortality and CVD events in several studies, including among the most recent works (21–25). Moreover, few prospective studies have also shown that decreased LTL predicts future development of type-2 diabetes (T2D) (26, 27).

In view of advances in tailored health-care management, in this study, we test the hypothesis that overweight and severe-obese subjects who carry an high number of CYP27A1 low-hydroxylation activity alleles (rs4674345 A, rs1554622 A, and rs4674338 G) are more likely to present altered cholesterol metabolism, premature aging at the cellular level as reflected in shorter LTL, as well as higher levels of CVD risk factors as compared to those with normal-hydroxylation activity. The aim is to identify persons more susceptible to develop CVD on which better converge preventive measures. To test this hypothesis, we genetically characterized overweight and severe-obese subjects of the SPHERE (“Susceptibility to Particle Health Effects, miRNAs and Exosomes”) project by the three functional polymorphisms of CYP27A1 (rs4674345 A/G, rs1554622 A/C, and rs4674338 G/A) and characterized each subject with a score ranging from 0 to 6, referring to the copy number of low-hydroxylation alleles. The combination of allele copy number and body mass index (BMI) categories was evaluated in relation to biological aging (LTL) and to well-known and novel CVD risk factors, such as inflammation and coagulation markers, and a CVD risk score [calculated according to the Cardiovascular Risk Calculator^1^].

## Materials and Methods

### Study Subjects

Study population consists of *n* = 1,457 participants enrolled in the SPHERE project.^2^ The recruitment was conducted at the Center for Obesity and Work, Fondazione IRCCS Ca’ Granda Ospedale Maggiore Policlinico, Milan, Italy period September 2010 and until March 2015. Each participant signed an informed consent form, which had been approved by the ethics committee of the Ca’ Granda Ospedale Maggiore Policlinico, Milan institution (approval number 1425), in accordance with principles of the Helsinki Declaration. The eligibility criteria for participants were as follows: (1) older than 18 years at enrollment; (2) obese/overweight according to the following definition: overweight is defined as a BMI between 25 and 30 kg/m^2^, obesity is defined as a BMI of 30 kg/m^2^ or more; (3) resident in Lombardy at the time of the recruitment; and (4) agreement to sign an informed consent and donate blood and urine samples. Exclusion criteria include the following: previous diagnosis of cancer, heart disease, or stroke in the last year or other chronic diseases such as multiple sclerosis, Alzheimer’s disease, Parkinson’s disease, depression, bipolar disorder, schizophrenia, and epilepsy (28, 29). Weight and height to calculate BMI were measured the same day of blood drawing. BMI was calculated as the weight in kilograms divided by the square of height in meters. The main characteristics of study participants are reported in Table 1. Most participants are females (72.75%).

**Table 1:** Demographic and lifestyle characteristics.

Characteristics	Categories	*n* = 1,457
Sex	Male	397 (27.25%)
	Female	1,060 (72.75%)

Age, years	Mean ± SD	52.26 ± 13.53

Body mass index (BMI), kg/m^2^	Mean ± SD	33.6 ± 5.4

BMI categorical	Overweight	959 (65.8%)
	Severe obese	498 (34.2%)

Waist to hip, cm	Mean ± SD	101.8 ± 13.2

Year of enrollment	2010	130 (8.92%)
	2011	408 (28.00%)
	2012	367 (25.19%)
	2013	313 (21.48%)
	2014	239 (16.4%)

Smoking	Never smoker	713 (49.00%)
	Ex-smoker	511 (35.12%)
	Actual smoker	231 (15.88%)

Cigarettes smoked^a^ (*n*/day)	≤5	65 (28.38%)
	5–10	59 (25.76%)
	10–15	42 (17.9%)
	15–20	48 (20.52%)
	20–40	17 (7.42%)

Pack/years (*n* = 1,380)	Median [IQR]	
	Among current and former smokers	15 [23–45]
	Including non-smokers	0 [14–25]

Time since quitting (*n* = 495)	Median [IQR]	13.14 [18–27]

Alcohol intake category (glasses)	Drink-unit/week (mean ± SD)	
	Wine	1.96 ± 3.76
	Beer	0.39 ± 0.99
	Hard liquor	0.23 ± 1.12

Physical activity	Sedentary	866 (59.4%)
	Active	457 (31.4%)
	Sporty	49 (3.4%)
	Active-sporty	44 (3.0%)
	Missing	41 (2.8%)

*^a^Among current smokers*.

*IQR = Q1 (first quartile)–Q3 (third quartile): interquartile range*.

### Epidemiological and Clinical Data Collection

As part of the routine protocol, for each subject presenting at the Center, an extensive physical examination was performed. Pulmonary functions were measured, at the same day of blood drawing, with an electronic flow volume spirometer V-max 22 with Autobox (SensorMedics), according to European Respiratory Society/American Thoracic Society guidelines (ERS/ATS 2005). Tests were performed on patients in the sitting position and were repeated until at least three reproducible forced expiratory curves were obtained. A resting electrocardiogram and rhythm strip were also recorded, and blood pressure was measured with the participant supine, after 5 min of rest. Whole blood was collected into ethylenediaminetetraacetic acid (EDTA) tubes from each participant by venous phlebotomy after overnight fast.

Biochemical parameters were also collected using standard procedures. Briefly, all patients underwent routine laboratory determinations, including complete blood counts (*n*/mm^3^), C-reactive protein (CRP; mg/dl), total-cholesterol (Total-C), high-density lipoprotein (HDL), and low-density lipoprotein (LDL), triglycerides, serum creatinine, fasting blood glucose, homocysteine, glycated hemoglobin, postprandial glycemia, insulin level, 2-h post glucose insulin level, urinary pH, and uric acid (28, 29). Quantitative determination of fibrinogen in citrate plasma samples was obtained on automated I.L. Coagulation System (Instrumentation Laboratory S.p.A., Milan, Italy). Insulin resistance was quantified by the homeostasis model assessment (HOMA) index using the formula described by Matthews et al. (30): HOMA index = [fasting serum insulin (mU/l) × fasting blood glucose] (mmol/l)/22.5. In addition, each study subject was asked to fill in a lifestyle questionnaire and to donate a 15 ml blood sample for molecular tests.

### Lifestyle Questionnaire

As previously described in Ref. (28), the lifestyle questionnaire collected information on sociodemographic data (sex and age) residential area (complete address, characteristics of the house, and traffic), education (i.e., primary school or less, high school, and university), smoking (i.e., never, former, and current smokers; cigarette smoked per day), history including passive smoking at home and at workplace, past and present health status of both the subjects and their first-degree relatives, medications in the last year, employment history (i.e., employee, unemployed, pensioner, and housewife) and address of the plant of their current work (currently employed subjects only), physical activity levels and sedentary behavior, commuting time, and transport mode. The questionnaire was self-assessed at recruitment and checked by a trained assistant, in presence of the subject, to ensure the completeness of information. Data originally recorded on paper (i.e., questionnaire) were then transferred into spreadsheets and anonymized of personally identifying information and identified through a five-digit randomly assigned barcode.

### Biological Sample Collection

Specific laboratory Standard Operating Procedures have been developed to ensure quality control of every step involved in biospecimen collection and storage. Blood was collected using EDTA tubes (7 ml). Blood samples were transported from the Center for Obesity and Work to EPIGET laboratory (University of Milan) within 2 h from the phlebotomy. EDTA blood was processed to obtain whole DNA from peripheral blood leukocytes. EDTA blood was centrifuged 1,100 × *g* for 15 min at room temperature to obtain platelet-free blood plasma and buffy coat. An aliquot of plasma was further centrifuged at 1,000, 2,000, and 3,000 × *g* for 15 min at 4°C to remove cell debris. Biospecimens were tracked through a secure database that stores detailed information on sample description, aliquoting, and freezer locations. Approximately 90% of study subjects donated a blood sample.

### Genotyping

DNA was isolated from frozen leukocytes as previously described in Ref. (29). The extracted DNA was stored at −20°C until shipping to the Department of Cardiac, Thoracic and Vascular Science, University of Padova, Italy. Genotyping was performed by commercially available TaqMan drug metabolism genotyping assays: C-2070266-20 “rs4674345” (Functionally Tested), C-2070139-10 “rs1554622” (Validated) and C-32303836-10 “rs4674338” (Functionally Tested) (Life Technologies–Thermo Fisher, Milan, Italy). Reactions were set up based on the manufacturer’s protocol, and the samples were run in triplicate on a Steponeplus Real-Time instrument (Applied Biosystems, Foster City, CA, USA). Allelic discrimination was performed using the SDS software v2.3 (Applied Biosystems). Twenty-five microlitre reactions in 96-well plates included 12.5 µl TaqMan Universal PCR Master Mix, No AmpErase UNG (2×), 1.25 μl Drug Metabolism Genotyping Assay Mix (20×) (Applied Biosystems), and DNA 11.25 μl (1 ng/μl). Validated Assays are tested using gDNA from 45 individuals from four ethnicities. The minor allele frequency for each population is available and is shown to be >5% in at least one population. Functionally Tested Assays met minimum functionality criteria in testing of 20 DNA samples from three ethnic populations and both genders. Minor allele frequency is not calculated. To find out whether the genotype was in Hardy–Weinberg equilibrium, distribution of the observed and expected genotype frequencies was compared using a chi-square test. For CYP27A1 genotype, values of 6, 5, 4, 3, 2 1, or 0 were attributed, referring to the presence of 6, 5, 4, 3, 2, 1, 0, copies of low hydroxylation (LH) activity rs4674345 A, rs1554622 A, rs4674338 G variants, respectively (as described in Table S1 in Supplementary Material) assuming the addictive effect of the presence of at least one allele at risk. In fact, each SNP showed similar contribution to CYP27A1 activity reduction that was 0.62, 0.65, and 0.60%, respectively (16). To detect the higher effect related to CYP27A1 reduced activity, we therefore considered >3 SNPs as cut point for statistical analysis. In this way, subjects with >3 LH alleles present at least a copy of each LH SNP.

### Cytokines Measurements

We used 50 µl plasma EDTA to quantify a custom panel of five cytokines using Luminex xMAP^®^-based technology (MYRIAD RBM, Inc., Austin, TX, USA), a multiplex immunoassay that simultaneously quantifies multiple protein analysts in a single run of the assay. In particular, we measured interferon gamma (IFNγ), macrophage inflammatory proteins alpha (MIPα), chemoattractant protein-1 (MCP-1), interleukin-10 (IL10), and tumor necrosis factor alpha (TNFα).

### Leukocyte Telomere Length (LTL) Measurement

Seven milliliters of whole blood were collected into EDTA tubes from each participant by venous phlebotomy after overnight fast. Blood was centrifuged at 2,500 rpm for 15 min. The buffy coat fraction was transferred to a cryovial and immediately frozen at −80°C until use. DNA was extracted by the Wizard Genomic DNA purification kit (Promega, Madison, WI, USA), according to the manufacturer’s instructions. LTL was measured by using quantitative Real-Time PCR method by Cawthon (31). This assay measures relative LTL in genomic DNA by determining, respectively, the ratio of telomere repeat copy number (T) to single nuclear copy gene (S), T/S ratio, in a given sample relative to a reference DNA. As reference DNA, we used a pool DNA from 50 participants randomly selected from the population (500 ng for each sample). The single-copy gene used in this study was human (beta) globin (hbg). A fresh standard curve, from the pool of control samples, ranging from 70 to 1.09 ng/µl (serial dilutions 1:2), was included in every “T” and “S” PCR run, against a negative control (water); 30 ng of DNA sample was added to each reaction. Each sample was run in triplicate. A high-precision MICROLAB STARlet Robot (Hamilton Life Science Robotics, Bonaduz AG, Switzerland) was used for transferring volume of 7 µl reaction mix and 3 µl DNA (10 ng/µl) in a 384-well format plate as previously described in Ref. (28, 29). All PCR reactions were performed on a 7900HT Fast Real Time PCR System (Applied Biosystems). The thermal cycling profile for both amplicons began with 50°C for 2 min followed by incubation at 95°C for 2 min to activate the AmpliTaq DNA polymerase. For telomere PCR, activation was followed by 2 cycles of 15 s at 95°C and 15 s at 49°C and 35 cycles of 15 s at 95°C, 10 s at 62°C, and 15 s at 74°C. For hbg, activation was followed by 35 cycles of 15 s at 95°C and 1 min at 58°C. At the end of each real-time PCR reaction, a melting curve was added for both T and S PCRs to verify the specificity of amplification. The average of the three T measurements was divided by the average of the three S measurements to calculate the average T:S ratio, i.e., relative telomere length. T:S ratio is normalized with T:S obtained from the reference pool of DNA inserted in each plate. A measure was considered acceptable if the SD among triplicate measures was <0.25. The coefficient of variation for the average T:S ratio of samples analyzed over three consecutive days was 8.7%, similar to the reproducibility originally reported for this method.

### Cardiovascular Risk Calculation

A 10-year cardiovascular risk score for estimating the probability of CVD was calculated by a Cardiovascular Risk Calculator, available at the web site http://cvrisk.mvm.ed.ac.uk/calculator/excelcalc.htm. This web page contains a downloadable version of the Framingham and ASSIGN scores, in an Excel spreadsheet, for use by persons wishing to calculate risk for large numbers of patients. The calculation takes into consideration age, sex, smoking behaviors (yes vs no), diabetes (yes: glycated hemoglobin ≥ 47.5 mmol/mol, no: glycated hemoglobin < 47.5 mmol/mol), systolic blood pressure (SBP), Total-C, and high-density lipoprotein-cholesterol (HDL-C).

### Statistical Analysis

Descriptive statistical data were obtained for the demographic/lifestyle variables and for CVD risk factors. Respectively, mean and SD were calculated for normally distribute data, median and interquartile range for non-normally distributed data. Number and percentage were reported for categorical variables. For CYP27A1 genotype, values of 6, 5, 4, 3, 2, 1, or 0 were attributed, referring to the presence of 6, 5, 4, 3, 2, 1, 0, copies of LH activity rs4674345 A, rs1554622 A, and rs4674338 G variants, respectively (as described in Table S1 in Supplementary Material) assuming the addictive effect of the presence of the allele at risk. In fact, each SNP showed similar contribution to CYP27A1 activity reduction that was 0.62, 0.65, and 0.60%, respectively (16). To detect the higher effect related to CYP27A1 reduced activity, we therefore considered more than three SNPs as cut point for statistical analysis. In this way, subjects with more than three LH alleles present at least a copy of each LH SNP.

The presence of 0–2 and 3–6 LH CYP27A1 alleles categorizes patients in LH: 0–2 and LH: 3–6 groups. We compare CVD risk factors of study participants according to BMI level: overweight (BMI < 35 kg/m^2^) vs severe-obese (BMI ≥ 35 kg/m^2^) using the χ^2^-test or Fisher’s exact test for categorical variables and the *t*-test or Wilcoxon rank-sum test for continuous variables.

Patients were then categorized in four BMI-CYP27A1 categories: overweight-LH: 0–2 the lowest risk group, overweight-LH: 3–6, severe-obese-LH: 0–2, and finally severe-obese-LH: 3–6, the highest risk group. In particular, the effects of genotype are studied in two different phenotypic background in following comparisons: overweight-LH: 3–6 vs overweight-LH: 0–2 and severe-obese-LH: 3–6 vs severe-obese-LH: 0–2. The effects of phenotype are studied in different phenotypic background with the same genotype, in the following comparison: severe-obese-LH: 0–2 vs overweight-LH: 0–2.

Simple univariate and multivariable linear regression models were carried out to evaluate the relationship between subjects belonging to the four CYP27A1 LH-BMI categories and CVD risk factors [Total-C, HDL-C, triglycerides, waist to hip, SBP, diastolic blood pressure (DBP), and heart rate], T2D (glycated hemoglobin ≥ 47.5 mmol/mol), biological aging (LTL), and inflammation and coagulation markers (CPR, fibrinogen, and leukocytes). Regression diagnostics were performed separately for each model, and outliers were excluded from regression analysis by dropping observations with studentized residuals that exceeded +3 or −3. The number of removed outliers ranged from a minimum of 0 to a maximum of 3.

We checked regression assumptions by performing diagnostic tests for each model, which included the Shapiro–Wilk test to verify normality of residuals and the White test to verify the homogeneity of variance of the residuals. Assumptions were satisfied for all continuous variables, except for HDL-C, LTL, CRP, leukocytes which were transformed on a logarithmic scale (log-transformed). The independent variables used in multivariable models were selected *a priori* and included general characteristics potentially associated with outcomes: sex, age, and smoking status (yes, no, and ex-smokers). For variables modeled on original scale, we calculate beta and 95% confidence intervals (CIs) from linear regression models, expressing the change in arithmetic mean of outcome switching from one category to the own reference one (overweight-LH: 0–2 or severe-obese-LH: 0–2). For log-transformed variables, we reported the % change = (exp(β) − 1) × 100 and 95% CIs expressing the percent change in geometric mean of variables switching from one category (overweight-LH: 3–6, severe-obese-LH: 3–6, and severe-obese-LH: 0–2) to the own reference one (overweight-LH: 0–2 or severe-obese-LH: 0–2).

Given the particular shape of cytokines variables (IFNγ, MIPα, MCP-1, IL10, and TNFα) distribution, which are all left-censoring because values that fall at or below some threshold are censored, we used univariate and multivariable Tobit regression adjusted for sex, age, smoking status (yes, no, and ex-smokers) to estimate the association between CYP27A1 LH-BMI categories and cytokines. Tobit regression is able to provide better consistent estimates of the parameters than the simple linear regression. Also in this case, we reported beta and 95% CIs, expressing the change in arithmetic mean of outcome switching from one category to the own reference one (overweight-LH: 0–2 or severe-obese-LH: 0–2).

Afterward, we computed odds ratios (ORs) and 95% CIs for the association of CYP27A1 LH-BMI category with insulin resistance (yes: 0 < HOMA index < 2.5; no: HOMA index > 2.5). Multivariable logistic regression models were adjusted for sex, age, and smoking status (yes, no, and ex-smokers). Finally, a univariate simple regression model was fitted to evaluate the association of CYP27A1 LH-BMI category with the 10-year cardiovascular risk score estimating the probability of CVD. Statistical analysis was carried out using SAS software (version 9.2; SAS Institute, Milan, Italy). A two-sided *p*-value of less than 0.05 was considered statistically significant.

## Results

Table 2 shows that quite all CVD risk factors such as classic modifiable risk factors (SBP and DBP), heart rate, lipid profile (Total-C, HDL-C, and triglyceride), glucose homeostasis (insulin resistance and glycated hemoglobin), and novel risk factors such as inflammation markers (leukocytes, CRP, MCP-1, and IL10), coagulation markers (fibrinogen), and biological aging indicator (LTL) were significantly (*p* < 0.001) different between overweight and severe-obese individuals.

**Table 2:** Cardiovascular disease and type-2 diabetes (T2D) risk factors according to body mass index levels.

Characteristics	All (1,457)	Overweight (*N* = 959)	Severe-obese (*N* = 498)	*p*-Value**
**Blood pressure, mmHg**				
Systolic blood pressure	125.8 ± 15.7	123.5 ± 14.9	130.3 ± 16.4	<0.001
Diastolic blood pressure	78.5 ± 9.36	77.4 ± 8.8	80.7 ± 10	<0.001
Above 140/90, mmHg	67 (4.60%)	24 (2.5%)	43 (8.6%)	0.2017
Below 140/90, mmHg	1,390 (95.40%)	935 (97.5%)	455 (91.4%)	–
Heart rate, bpm	67.6 ± 10.5	66.3 ± 10.1	69.9 ± 11	<0.001
Total-cholesterol, mg/dl	212.6 ± 40.4	213.1 ± 39.9	211.5 ± 41.3	0.4898
High-density lipoprotein-cholesterol, mg/dl	57 [20]	59 [22]	54 [17]	<0.001
Low-density lipoprotein-cholesterol, mg/dl	133.6 ± 35.4	133.2 ± 35.3	134.4 ± 35.4	0.5314
Triglycerides, mg/dl	107 [67]	99 [67]	118 [73]	<0.001
Total-cholesterol/high-density lipoprotein-cholesterol	3.83 ± 1.2	3.7 ± 1.2	4.0 ± 1.2	<0.001
Glucose, mg/dl	98.6 ± 24.9	96.4 ± 22.4	102.9 ± 28.7	<0.001
Glycated hemoglobin, mmol/mol	40.3 ± 8.6	39.2 ± 7.6	42.3 ± 10.0	<0.001
Insulin level, mIU/L	14.9 ± 11.4	12.7 ± 8.5	19.1 ± 14.7	<0.001

**Type-2 diabetes^b^**				
Yes	171 (11.9%)	84 (8.9%)	87 (17.7%)	<0.001
No	1,270 (88.1%)	864 (91.1%)	406 (82.4%)	–

**Insulin resistance^c^**				
Yes	774 (54.9%)	425 (46.1%)	349 (71.5%)	<0.001
No	637 (45.2%)	498 (54.0%)	139 (28.5%)	–

Complete blood count (n*10^3^/µl)				
Leukocytes	6.65 [2.24]	6.44 [2.16]	7.01 [2.26]	<0.001
Red blood cells	4.8 ± 0.5	4.8 ± 0.5	4.8 ± 0.4	0.0652
Mean corpuscolar colume	85.3 ± 7.6	85.4 ± 8.2	85.1 ± 6.2	0.3336
Platelets	248.9 ± 59.5	246.6 ± 56.8	253.3 ± 64.1	0.0497

**Inflammation markers**				
C-reactive protein, mg/dl	0.28 [0.42]	0.22 [0.31]	0.46 [0.57]	<0.001

**Cytokines**				
IFNγ, pg/ml	9.3 [12.35]	9.2 [11.35]	9.3 [12.35]	0.2018
MIPα, pg/ml	68.0 [75]	67 [76]	70 [76]	0.1885
MCP-1, pg/ml	169.0 [82]	165 [79]	174 [85]	<0.001
IL10, pg/ml	3.7 [2.7]	3.6 [2.7]	4.1 [2.8]	<0.001
TNFα, pg/ml	5.5 [5.8]	5.05 [5.6]	6.3 [6.4]	0.0305

**Coagulation markers**				
Fibrinogen, mg/dl	331.2 ± 63.3	321.7 ± 59.8	349.8 ± 65.8	<0.001

**Biological aging**				
LTL (T/S)	1.05 ± 0.30	1.1 ± 0.3	1.0 ± 0.3	<0.001

**Genotype** **CYP27A1 LH group^a^**				
LH: 0–2	358 (24.6%)	234 (24.4%)	124 (24.9%)	0.8337
LH: 3–6	1,099 (75.4%)	725 (75.59%)	374 (75.1%)	–

*Variables were expressed as mean ± SD and median [IQR] if continuous and *N* (%) as categorical*.

**p-Value from t-test or Wilcoxon Rank sum test for continuous variables and χ^2^ for categorical variables.

*^a^Presence of 0–2 and 3–6 low-hydroxylation (LH) alleles (rs4674345 A, rs1554622 A, and rs4674338 G) categorizes patients in LH: 0–2 and LH: 3–6 groups*.

*^b^Type-2 diabetes (yes: glycated hemoglobin ≥ 47.5 mmol/mol; no: glycated hemoglobin < 47.5 mmol/mol)*.

*^c^Insulin resistance [yes: 0 < homeostasis model assessment (HOMA) index < 2.5; no: HOMA index > 2.5]*.

Univariate unadjusted and multivariable sex–age–smoking-adjusted linear regression models are presented in Tables 3 and 4.

**Table 3:** Univariate analysis.

CYP27A1 LH-BMI category												
Variables	Overweight-LH: 3–6 vs overweight-LH: 0–2				Severe-obese-LH: 0–2 vs overweight-LH: 0–2				Severe-obese-LH: 3–6 vs severe-obese LH: 0–2			
	Effect^a^	95% CI		*p*-Value	Effect	95% CI		*p*-Value	Effect	95% CI		*p*-Value
**Cardiovascular disease risk factors**												
Total-cholesterol	4.40	−1.58	10.37	0.1490	−1.45	−10.26	7.36	0.7468	4.28	−3.94	12.50	0.3073
High-density lipoprotein-cholesterol^b^	−3.50	−7.12	0.26	0.0679	−9.45	−14.42	−4.20	<0.001	−0.58	−5.68	4.79	0.8283
Triglycerides^b^	4.08	−2.89	11.55	0.2576	18.40	6.90	31.13	0.0012	1.10	−8.09	11.22	0.8212
Waist to hip	0.23	−1.30	1.77	0.7650	17.53	15.26	19.80	<0.001	−0.22	−2.33	1.90	0.8396
Systolic blood pressure	0.87	−1.41	3.14	0.4557	7.74	4.36	11.12	<0.0001	−0.41	−3.56	2.75	0.7997
Diastolic blood pressure	0.52	−0.84	1.89	0.4516	4.31	2.28	6.33	<0.0001	−0.78	−2.67	1.11	0.4164
Heart rate	0.71	−0.83	2.25	0.3628	4.00	1.72	6.28	0.0006	0.15	−1.98	2.27	0.8923

**Type-2 diabetes**												
Glycated hemoglobin^b^	0.28	−2.35	2.99	0.8355	8.97	4.78	13.31	<0.001	1.88	−5.34	1.83	0.3243

**Oxidative stress**												
LTL^b^	0.43	−3.87	4.91	0.8486	0.38	−5.90	7.08	0.9089	−5.61	−11.13	0.25	0.0602

**Inflammation and coagulation markers**												
C-reactive protein^b^	−6.54	−19.47	8.46	0.3727	62.04	30.17	101.72	<0.0001	19.40	−2.66	46.43	0.0888
Fibrinogen	−5.41	−14.65	3.84	0.2515	28.42	14.62	42.22	<0.0001	−5.76	−18.68	7.16	0.3819
Leukocytes^b^	−0.02	−0.05	0.02	0.3474	0.07	0.02	0.12	0.0096	1.52	−3.45	6.74	0.5560

**Cytokines^c^**												
MCP-1	2.51	−4.29	9.79	0.4789	12.63	1.72	24.69	0.0221	0.64	−8.47	10.64	0.8957

	**OR^d^**	**95% CI**		***p*-Value**	**OR^d^**	**95% CI**		***p*-Value**	**OR^d^**	**95% CI**		***p*-Value**

Insulin resistance (yes vs no)	1.49	1.10	2.01	<0.001	3.81	2.36	6.14	0.0022	1.37	0.86	2.20	<0.0001

*^a^For variables in the original scale, we reported as effect the coefficient beta and 95% confidence intervals (CIs) expressing the change in arithmetic mean of outcome switching from one category to the own reference one [overweight-low hydroxylation (LH): 0–2 or severe-obese LH: 0–2]*.

*^b^For log-transformed variables, we reported as effect Δ% = (exp(β) − 1) × 100 and 95% CIs expressing the percent change in geometric mean of variables switching from one category to the own reference one (overweight-LH: 0–2 or severe-obese-LH: 0–2)*.

*^c^Cytokines were modeled by means of Tobit regression. Also in this case, we reported as effect the coefficient beta and 95% CIs, expressing the change in arithmetic mean of outcome switching from one category to the own reference one (overweight-LH: 0–2 or severe-obese-LH: 0–2)*.

*^d^Odds ratio (OR) and 95% CIs from adjusted logistic regression for sex, age, and smoking*.

The effects of genotype (LH: 3–6 vs LH: 0–2 with the same phenotypic conditions) were studied in the comparisons overweight-LH: 3–6 vs overweight-LH: 0–2 and severe-obese-LH: 3–6 vs severe-obese-LH: 0–2. Multivariable sex–age–smoking-adjusted (Table 4) shows that severe-obese-LH: 3–6 present higher LTL attrition (β = −5.86; *p* = 0.039) than severe-obese-LH: 0–2. Univariate unadjusted and multivariable sex–age–smoking-adjusted linear regression models (Tables 3 and 4) show that insulin resistance was higher in overweight-LH: 3–6 vs overweight-LH: 0–2 (OR = 1.34; *p* < 0.001) and in severe-obese-LH: 3–6 vs severe-obese-LH: 0–2 (OR = 1.41; *p* < 0.0001). Moreover, HDL-C was quite significantly reduced (β = −3.50, *p* < 0.0679) in overweight-LH: 3–6 vs overweight-LH: 0–2 (Table 3). The unadjusted and sex–age–smoking-adjusted regression diagnostic models we applied for this borderline difference (data not showed) indicated that HDL-C was significantly reduced in overweight-LH: 3–6 vs overweight-LH: 0–2 [β = −4.16 (95% CI = −7.60; −0.61, *p* = 0.0223) and β = −3.89 (95% CI = −6.9; −0.72, *p* = 0.0167)].

**Table 4:** Multivariable analysis.

CYP27A1 LH-BMI category												
Variables	Overweight-LH: 3–6 vs overweight-LH: 0–2				Severe-obese-LH: 0–2 vs overweight-LH: 0–2				Severe-obese-LH: 3–6 vs severe-obese-LH: 0–2			
	Effect^a^	95% CI		*p*-Value	Effect	95% CI		*p*-Value	Effect	95% CI		*p*-Value
**Cardiovascular disease risk factors**												
Total-cholesterol	3.89	−1.99	9.77	0.1947	−1.53	−10.19	7.14	0.7296	3.68	−4.39	11.75	0.3711
High-density lipoprotein-cholesterol^b^	−2.85	−6.16	0.57	0.1014	−8.28	−12.84	−3.48	<0.001	−1.36	−5.94	3.44	0.5709
Triglycerides^b^	2.85	−3.84	10.00	0.4131	16.26	5.30	28.36	0.0029	1.58	−7.38	11.39	0.7394
Waist to hip	−0.25	−1.51	1.02	0.7020	16.42	14.56	18.28	<0.001	0.13	−1.61	1.86	0.8835
Systolic blood pressure	0.36	−1.80	2.51	0.7467	6.76	3.56	9.96	<0.0001	-0.20	−3.19	2.78	0.8952
Diastolic blood pressure	0.36	−0.98	1.71	0.5968	3.95	1.95	5.94	0.0001	−0.66	−2.52	1.20	0.4877
Heart Rate	0.88	−0.66	2.41	0.2622	4.38	2.12	6.64	0.0002	0.03	−2.08	2.14	0.9760

**Type-2 diabetes**												
Glycated hemoglobin^b^	−0.05	−2.53	2.49	0.9695	8.21	4.29	12.28	<0.001	−1.83	−5.15	1.60	0.2910

**Oxidative stress**												
LTL^b^	1.16	−2.97	5.47	0.5874	1.99	−4.10	8.46	0.5306	−5.86	−11.10	−0.31	0.039

**Inflammation and coagulation markers**												
C-reactive protein^b^	−5.62	−18.61	9.44	0.4436	65.32	32.99	105.48	<0.0001	18.41	−3.30	45.01	0.1019
Fibrinogen	−5.90	−15.04	3.25	0.206	29.48	15.84	43.11	<0.0001	−7.07	−19.81	5.68	0.2770
Leukocytes^b^	−2.17	−5.57	1.36	0.2246	7.75	2.28	13.51	0.005	1.30	−3.50	6.34	0.6027

**Cytokines^c^**												
MCP-1	1.72	−4.91	8.81	0.6192	10.84	0.30	22.47	0.0435	0.64	−8.28	10.42	0.8933

	**OR^d^**	**95% CI**		***p*-Value**	**OR^d^**	**95% CI**		***p*-Value**	**OR^d^**	**95% CI**		***p*-Value**

Insulin resistance (yes vs no)	1.34	0.97	1.84	<0.001	3.42	2.09	5.61	0.0036	1.41	0.88	2.27	<0.0001

*^a^For variables in the original scale, we reported as effect the coefficient beta and 95% confidence intervals (CIs) resulting from adjusted linear regression models [adjusted for sex, age, and smoking status (yes, no, and ex-smokers)], expressing the change in arithmetic mean of outcome switching from one category to the own reference one [overweight-low hydroxylation (LH): 0–2 or severe-obese-LH: 0–2]*.

*^b^For log-transformed variables, we reported as effect Δ% = (exp(β) − 1) × 100 and 95% CIs expressing the percent change in geometric mean of variables switching from one category to the own reference one (overweight-LH: 0–2 or severe-obese-LH: 0–2)*.

*^c^Cytokines were modeled by means of Tobit regression. Also in this case, we reported as effect the coefficient beta and 95% CIs, expressing the change in arithmetic mean of outcome switching from one category to the own reference one (overweight-LH: 0–2 or severe-obese-LH: 0–2)*.

*^d^Odds ratio (OR) and 95% CIs from adjusted logistic regression for sex, age, and smoking*.

The effects of phenotype (severe-obese vs overweight with the same genotype), studied in the comparison severe-obese-LH: 0–2 vs overweight-LH: 0–2, are evident in most (71%) of the well-known (blood pressure, heart rate, waist to hip, triglycerides, and HDL-C) and novel CVD risk factors [inflammation markers (CRP, leukocytes, and MCP-1), fibrinogen and glucose homeostasis (insulin resistance and glycated hemoglobin)], but not in LTL. The results point to the fact that obesity leads to worsen CVD risk factor profile.

**Figure 1:**
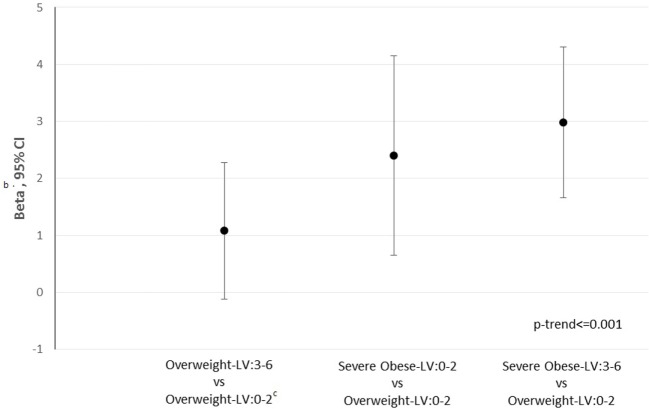
10-Year cardiovascular disease (CVD)^a^ risk according to CYP27A1 body mass index-low hydroxylation (LH) category. ^a^10-Year CVD score calculated using Framingham equation. ^b^We reported as effect the coefficient beta and 95% confidence intervals from adjusted linear regression models [adjusted for sex, age, smoking, status (yes, no and ex-smokers)], expressing the change in arithmetic mean of outcome switching from one category to the reference one (overweight-LH: 0–2). ^c^Reference: overweight-LH: 0–2.

In the whole, the 10-year CVD risk (Figure 1) raises from overweight–LH: 3–6 (β: 1.08, 95% CI: −0.1 to 2.28, *p* = 0.0754), severe-obese–LH: 0–2 (β: 2.40, 95% CI: 0.64–4.15, *p* < 0.0076), and even more in severe-obese-LH: 3–6 (β: 2.98, 95% CI: 1.66–4.30, *p* < 0.001) compared with the reference (overweight-LH: 0–2).

## Discussion

Our study, exploring the hypothesis that overweight and severe-obese individuals, who carry more than three LH cholesterol CYP27A1 variants (rs4674345 A, rs1554622 A, and rs4674338 G; LH: 3–6 group), are more likely to present higher CVD risk factors and premature biological aging, founds that LTL attrition and insulin resistance were significant higher in severe-obese-LH: 3–6 than severe-obese-LH: 0–2, while insulin resistance was also greater in overweight-LH: 3–6 compared with overweight-LH: 0–2. In the entire sample population, LTL was significant lower in severe-obese than overweight.

Moreover classic CVD risk factors (blood pressure, heart rate, waist to hip, triglycerides, and HDL-C) and novel CVD/T2D risk factors [inflammation markers (CRP, leukocytes, and MCP-1), fibrinogen, and glucose homeostasis (insulin resistance and glycated hemoglobin)] were significant altered in severe-obese vs overweight with the same genotype (LH: 0–2) point to the fact that obesity represents a harmful condition that leads to worsen the CVD risk factor profile.

### LTL and Obesity

Several observational studies have investigated the association between obesity and LTL (19, 20). Even if this association was found to be from weak to moderate and many studies did not reach statistical significance, there was a trend toward an inverse correlation between TL and obesity (19, 20). The only study that reported a positive association was conducted in women with systemic lupus erythematosus, and the authors state that this result might simply reflect a better disease control (32). Sex (33), ethnicity (34), and age (35) seem to be the main factors that mine this moderate association. The chances that the association—BMI with shorter LTL—could depend on factors other than BMI were minimized in our study because there is a prevalence of women, quite of the same age range and all are of the same ethnicity. The fact that, in the subgroup of CYP27A1 LH: 0–2 (*n* = 358), LTL was not significant in severe-obese vs overweight, it can be attributed to the lowered sample size of population.

Among the key factors that may explain the association between telomere attrition and obesity there are increased inflammatory processes linked to the oxidative stress that accompanies obesity (36). Obesity increases a pro-inflammatory state linked to hyperplasia and hypertrophy of adipocytes, and to adipose tissue hypoxia (37). This process likely induces increased production of adipokines, leading to a localized and systemic pro-inflammatory state with greater production of reactive species (38). Telomeres, as triple-guanine-containing sequences, are highly sensitive to damage by oxidative stress (39). The resulting damaged telomeric bases by double-strand breaks and/or interference with replication fork can induce a reduction in telomere length (40). Long-standing exposure to these inflammatory conditions give rise to significant changes also in large and microscopic blood vessels, increased leukocyte adhesion, thrombus formation, and impaired vasomotor responses (41, 42). Telomere erosion in peripheral blood leukocytes, the biological aging clock, is accelerated by a local and systemic status of inflammation (43, 44), representing a fundamental mechanism linking obesity with related diseases, such as CVD. Coherently in our study, inflammation (CRP, leukocytes, and MCP-1) and coagulation markers (fibrinogen) were significantly higher in severe-obese than overweight subjects. The clear significant association between increased BMI and LTL attrition adds further dowel which remarks the importance of obesity as a determinant of aging.

### Low-Hydroxylation CYP27A1 Variants in Severe Obese and Shorter LTL

Sterol 27-hydroxylase is involved in cholesterol efflux, as it represents the first and rate-limiting step in reverse transport, by transforming cholesterol to 27OHC in extrahepatic tissues. CYP27A1 is in fact expressed at high levels in the macrophages and vascular endothelium (45). Low activity of CYP27A1 results in shoddier cholesterol efflux (46). In fact, patients with cerebrotendinous xanthomatosis, lacking of CYP27A1, due to the deleterious effect of gene deletion mutations, present cholesterol abnormally deposited in different tissues, such as vascular endothelium, central nervous system, and crystalline lens (47). In addition, these patients significantly present low levels of HDL-C and are predisposed to develop premature aging-related diseases, including atherosclerosis and coronary heart disease (48). Our findings that LTL are lowered in the LH: 3–6 than LH: 0–2 severe-obese patients would suggest that telomere erosion is further accelerated by local and systemic status of inflammation as a consequence of the reduced cholesterol removal from peripheral tissues. This points out the importance by these CYP27A1 variants in identify, among severe-obese, those that present higher biological aging and are therefore at higher risk of developing premature aging-related diseases, than those without these harmful variants. The influence we found of CYP27A1 polymorphism on these fundamental inflammatory players in obesity, including LTL, would underline the importance of this enzyme not only in cholesterol removal, *via* reverse transport, but also in the consequent aging response.

### Low-Hydroxylation CYP27A1 Variants and Higher Insulin Resistance

The risk of being insulin resistance (HOMA index < 2.5) is evident among severe-obese and overweight individuals too with low-hydroxylation CYP27A1 polymorphism. Insulin resistance is a feature of T2D and is also implicated in obesity and in CVD development (49). The compensatory hyperinsulinemia due to insulin resistance induces free fatty acid efflux from adipose tissue, raises plasma triacylglycerol but reduces HDL-C, by activation of cholesterol ester transfer protein that increases its clearance by the kidneys (50, 51). Actually, in our study, we found that HDL-C was significantly reduced in overweight-LH: 3–6 vs overweight-LH: 0–2. Likewise, it is the loss of insulin sensitivity that explains why obese individuals are more likely to develop T2D and CVD, but not all overweight/obese individuals present insulin resistance. In light of these observations, it seems reasonable to suggest that insulin resistance is the link between overweight/obesity and the adverse clinical syndromes related to excess adiposity. Therefore, it is important to identify those overweight/obese individuals who are also insulin resistance, to initiate the most intensive therapeutic effort in this subgroup. The observation that the risk of being insulin resistance is higher among severe-obese and overweight individuals with low-hydroxylation CYP27A1 activity, open new perspectives in examining persons at higher risk to develop T2D and CVD, representing a new possible target/challenge in T2D and CVD prevention research.

### Low-Hydroxylation CYP27A1 Variants in Overweight Reduces HDL-C

HDL-C, which we found reduced in overweight LH: 3–6 compared with overweight LH: 0–2, represents the most important system of cholesterol removal from tissues, transporting it back (“reverse transport”) to the liver, where cholesterol is processed for excretion through the bile (52). Low HDL-C levels promote atherogenesis and increase CVD risk. Macrophages are the principal artifice of cholesterol efflux *via* the reverse transport (53). Patients with cerebrotendinous xanthomatosis significantly present low levels of HDL-C and are predisposed to develop cholesterol accumulation diseases, including atherosclerosis, coronary heart disease, cataracts, tendon xanthomas as well as the presence of progressive neurologic dysfunction (48). Low HDL-C levels have also been implicated in modifying survival and in the onset of respiratory impairment in amyotrophic lateral sclerosis patients (54). Since we found that HDL-C levels are lowered in the LH: 3–6 group, our study would suggest the importance also of these CYP27A1 variants in regulating cholesterol metabolism/efflux on the risk to develop “cholesterol accumulation diseases,” CVD included.

### Low-Hydroxylation CYP27A1 Variants and 10-Year Cardiovascular Risk Score

Likewise, the 10-year cardiovascular risk score, which estimates the 10-year probability of CVD in individuals who have not already developed major atherosclerotic disease, raises in overweight and even more in severe-obese subjects with low-hydroxylation cholesterol CYP27A1 variants. This score is considered an aid to make clinical decisions about how intensively to intervene on lifestyle and whether to use antihypertensive, lipid lowering medication and aspirin. The study’s findings, therefore, support the hypothesis that the CYP27A1 genetic characterization better identifies persons at higher risk to develop CVD on which better converge preventive measures, representing a new possible target in personalized medicine.

### Conclusion

Our research on CYP27A1 polymorphism opens new perspectives in investigation of the adverse health effects of obesity and in identifying persons at higher risk to develop T2D and CVD on which better converge preventive measures. CYP27A1 has the potential to act both as susceptibility marker in T2D and CVD development and as a key event in the chain of events connecting obesity to T2D and CVD development, representing a new possible target in personalized preventive medicine of chronic degenerative diseases. In addition, the clear significant role of CYP27A1 on the relationship between BMI and LTL attrition adds further insight into mechanism linking obesity with aging.

## Ethics Statement

The ethics committee of the Ca’ Granda Ospedale Maggiore Policlinico, Milan Institution (approval number 1425).

## Author Contributions

SP designed the study. LA and AP supervised the epidemiological design and performed bioinformatics analysis. SP, LA, MC, MH, and LC performed the laboratory experiments. LA, VB, and SP analyzed the data. AT was responsible for blood clinical analyses. LV recruited study subjects. SP, VB, and MH wrote the manuscript. All the authors contributed to the data interpretation and manuscript revision; read and approved the final manuscript.

## Conflict of Interest Statement

The authors declare that the research was conducted in the absence of any commercial or financial relationships that could be construed as a potential conflict of interest.

## Footnotes

^1^http://cvrisk.mvm.ed.ac.uk/calculator/excelcalc.htm (Accessed: November 11, 2016).

^2^http://users.unimi.it/sphere/ (Accessed: May 18, 2016).

## Abbreviations

27OHC, 27-hydroxycholesterol; BMI, body mass index; CRP, C-reactive protein; CVD, cardiovascular disease; T2D, type-2 diabetes; CYP27A1, sterol 27-hydroxylase; DBP, diastolic blood pressure; ECG, electrocardiogram; ERs, estrogen receptors; HDL-C, high-density lipoprotein-cholesterol; HOMA, homeostasis model assessment; IFNγ, interferon gamma; IL10, interleukin-10; LDL, low-density lipoprotein; LH, low-hydroxylation activity; LTL, leukocyte telomere length; MCP-1, chemoattractant protein-1; MIPα, macrophage inflammatory proteins alpha; SBP, systolic blood pressure; TNFα, tumor necrosis factor alpha; Total-C, total-cholesterol.
