# Supplementary material for: Sterol 27-Hydroxylase Polymorphism Significantly Associates With Shorter Telomere, Higher Cardiovascular and Type-2 Diabetes Risk in Obese Subjects
Source: Front Endocrinol (Lausanne). 2018 Jun 13;9:309. doi: 10.3389/fendo.2018.00309 (PMC6008574; doi:10.3389/fendo.2018.00309)
Supplement: Supplementary file 1 [file table_1.PDF]

Table1s Number of low hydroxylation alleles in relation to the presence of low activity SNPs.

Number of low hydroxylation alleles	**rsG4674345A**	**rsC1554622A**	**rsA4674338G**
6	**AA**	**AA**	**GG**
5	**A**G	**AA**	**GG**
	**AA**	**A**C	**GG**
	**AA**	**AA**	**G**A
4	**A**G	**A**C	**GG**
	**A**G	**AA**	**G**A
	**AA**	**A**C	**G**A
3	**A**G	**A**C	**G**A
2	GG	**A**C	**G**A
	**A**G	CC	**G**A
	**A**G	**A**C	AA
1	**A**G	CC	AA
	GG	**A**C	AA
	GG	CC	**G**A
0	GG	CC	AA
